# Treatment of a Post-biopsy Pulmonary Artery Pseudoaneurysm

**DOI:** 10.7759/cureus.21411

**Published:** 2022-01-19

**Authors:** Tatiana Melo, Pedro Pereira, João André Oliveira

**Affiliations:** 1 Imaging, Hospital do Espírito Santo de Évora, Évora, PRT; 2 Radiology, Centro Hospitalar Universitário do Porto, Porto, PRT

**Keywords:** systemic anticoagulation, thrombin, percutaneous treatment, pseudoaneurysm, pulmonary arteries

## Abstract

Peripheral pulmonary artery pseudoaneurysms (PPAP) following a lung biopsy are exceedingly rare but can lead to severe haemoptysis. Cases requiring treatment are usually managed using an endovascular approach. Nevertheless, successful percutaneous treatment has been described. Several embolic agents can be used to accomplish percutaneous embolization. We aim to report a successful percutaneous treatment of a post-biopsy PPAP using thrombin. Our patient developed a PPAP, with consequent large alveolar haemorrhage and haemoptysis, after a transthoracic lung biopsy. Because the patient had a mechanical mitral valve, she needed to be anticoagulated, impairing the spontaneous resolution of the pseudoaneurysm. Therefore, the PPAP had to be treated to safely anticoagulate the patient. A percutaneous approach was chosen given the very peripheric location of the PPAP. Treating PPAP with an endovascular approach can be challenging. With this report, we demonstrate that the percutaneous approach is a safe and efficient alternative method. We choose thrombin as an embolic agent given its safety profile and efficacy under anticoagulation. The good results of our intervention reinforce the applicability and efficacy of this kind of treatment approach to PPAP.

## Introduction

Pseudoaneurysms of the peripheral pulmonary artery are rare. These are most often associated with the use of Swan-Ganz catheters and cavitary tuberculosis [1,2]. Although uncommon, post-lung biopsy peripheral pulmonary artery pseudoaneurysms (PPAP) have also been described [3]. Owing to their uncontained nature, leakage of blood to the bronchial tree frequently occurs, causing haemoptysis that can be life-threatening [4].

Arterial pseudoaneurysms occur as a result of an injury to the vessel. Several risk factors have been associated with an increased risk for the formation of PPAP, namely, anticoagulation, which diminishes the ability of the artery to seal itself after injury [1]. Historically, surgical treatment was the gold standard for PPAP. However, advances in endovascular therapies have allowed good results for transcatheter embolization of pseudoaneurysms, which is a much less invasive option than surgery. Nowadays, endovascular treatment is considered the first-line treatment [1]. Endovascular treatment is usually achieved with coil embolization of the pulmonary artery branch feeding the aneurysmal sac [1]. Filling the aneurismal sac itself with coils is an alternative, as so the use of covered stents, epoxy glues, polyvinyl alcohol, and gelatine sponge pledgets [1].

## Case presentation

A 66-year-old woman treated seven years ago for breast carcinoma presented with multiple pulmonary nodules. During follow up, some of these nodules increased dimensions, while others decreased, so the diagnosis of metastatic disease was not straightforward. Additionally, in the setting of metastatic breast cancer, receptors had to be characterized. A transthoracic lung biopsy was requested. The patient was under chronic anticoagulation for a mechanical mitral valve.

**Figure 1:**
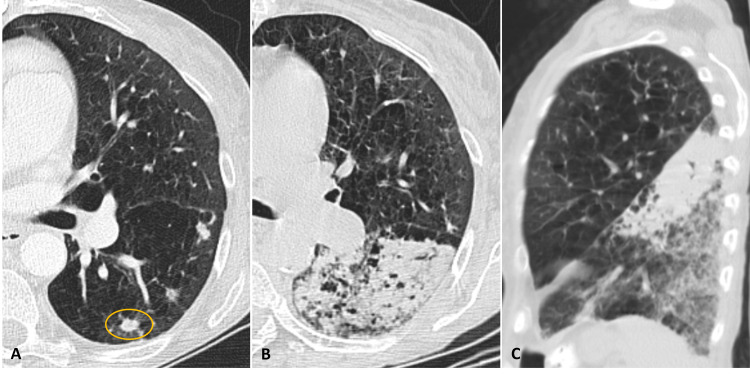
Pulmonary nodule and alveolar haemorrhage. Pulmonary window CT images. (A) Axial plane showing the subpleural nodule (circle) in the posterior segment of the inferior left pulmonary lobe before the biopsy. (B) Axial plane the day after the biopsy demonstrating the large alveolar haemorrhage. (C) Coronal reformation allows better evaluation of the extent of the alveolar haemorrhage, occupying almost the entire left lower lobe.

**Figure 2:**
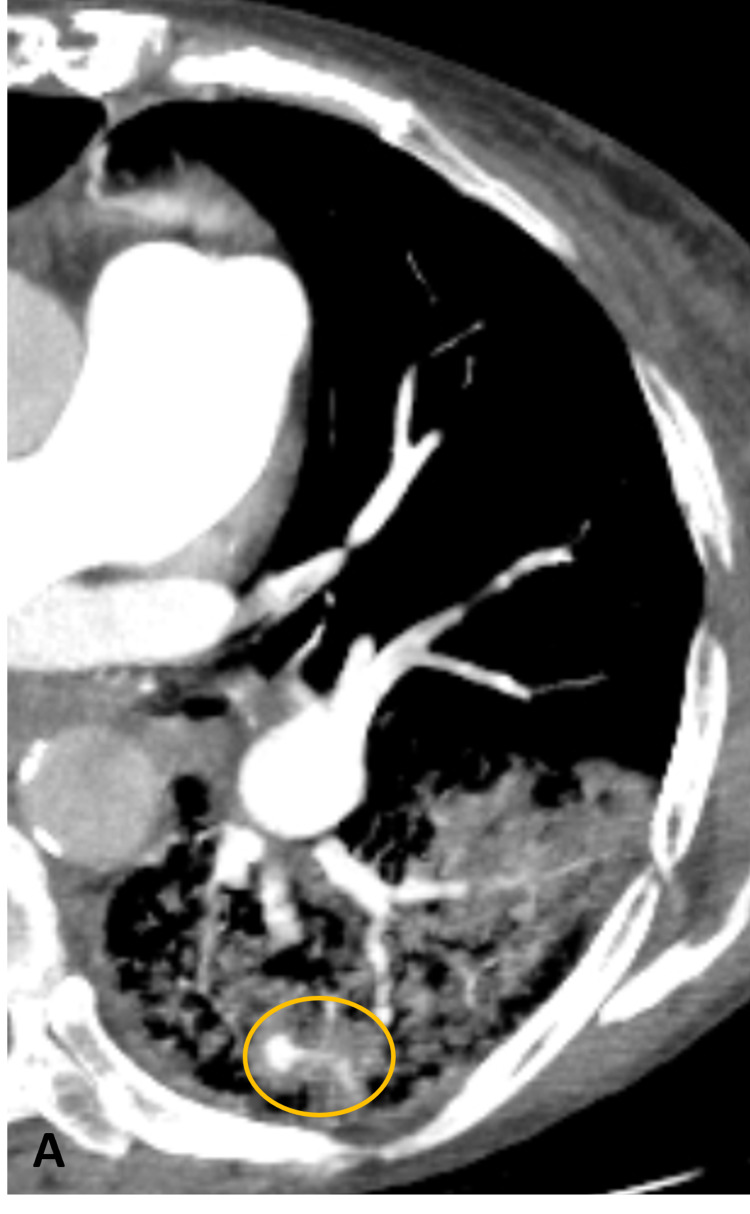
Peripheral pulmonary artery pseudoaneurysm. Five days post-biopsy CT image. Contrast-enhanced axial image at the level of the biopsy showing persistent alveolar haemorrhage and a subpleural peripheral pulmonary artery pseudoaneurysm (circle) at the site of the pulmonary nodule biopsy.

After careful revision of CT images, we decided to biopsy a 10-mm nodule located in the posterior segment of the inferior left pulmonary lobe (ILL) (Figure 1A). Biopsy was performed using a co-axial system for a 20G Tru-Cut needle, more than 24 hours after the last dose of enoxaparin. Three samples were collected. By the end of the biopsy, a large alveolar haemorrhage had developed, and the patient was experiencing haemoptysis. Unenhanced CT re-evaluation was performed the following day, revealing enlargement of the alveolar haemorrhage, occupying almost the entire ILL (Figures 1B, 1C). However, haemoptysis had ceased with the use of aminocaproic acid, and the patient was clinically stable, so anticoagulation was reinitiated the day after. Two days later, the patient developed new episodes of mild haemoptysis and dyspnoea requiring supplementary oxygen. A CT angiography was then performed, revealing a small pseudoaneurysm of a peripheral pulmonary artery branch near the biopsied nodule (Figure 2). Anticoagulation was once again withdrawn.

**Figure 3:**
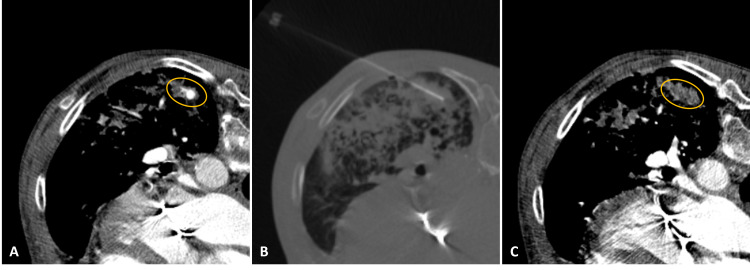
Percutaneous embolization treatment. (A) Post-contrast axial CT image with the patient in the prone position demonstrating the body of the pseudoaneurysm (circle). (B) Same axial CT plane confirming the correct position of the injection needle. (C) Control post-contrast axial CT image showing complete thrombosis of the pseudoaneurysm (circle) after percutaneous injection of thrombin.

Because of the mechanical mitral valve, the patient had to be anticoagulated, which impaired spontaneous resolution of the pseudoaneurysm. Therefore, it was decided to treat the pseudoaneurysm to safely anticoagulate the patient. Since it was a very peripheral arterial branch, we considered it to be best suitable for percutaneous rather than endovascular treatment. Under CT guidance, percutaneous puncture of the pseudoaneurysm sac was performed with a 22G needle (Figures 3A, 3B). Injection of 1000 U of thrombin was performed. CT angiography confirmed complete thrombosis of the pseudoaneurysm (Figure 3C). The patient started prophylactic anticoagulation the following day, without recurrence of the haemoptysis.

## Discussion

Cope and Zeit [5] first described direct percutaneous treatment with thrombin injection of pseudoaneurysms in 1986. In 2020, Lal et al. [6] published a series of PPAP successfully treated with percutaneous transthoracic embolization. In this paper, the authors concluded it to be a treatment option especially for peripherally located pseudoaneurysms [6]. In our case, the PPAP occurred in a subpleural location, so we considered it to be suitable for percutaneous rather than endovascular treatment.

Percutaneous embolization of pseudoaneurysms can be accomplished with several agents, such as ethylene vinyl alcohol (EVOH) copolymer, N-butyl cyanoacrylate, thrombin or even with coils.

Thrombin originates from prothrombin and has multiple roles in the blood clotting cascade. The administration of exogenous thrombin causes rapid activation of endogenous fibrinogen into fibrin, promoting fast thrombus formation [7]. Inside the pseudoaneurysm, there is relative blood stasis, so thrombin is less readily washed away, facilitating thrombus formation [8].

The use of bovine thrombin for pseudoaneurysm treatment is associated with a low complication rate (about 0-4%) [8]. The most frequently reported complication is distal embolization [8]. To prevent these, the injection should be performed slowly into the sac of the pseudoaneurysm, avoiding injecting the neck of the pseudoaneurysm [8]. As stated before, there is relative blood stasis in the pseudoaneurysm, which also diminishes the risk. Other less frequent reactions include adverse immunologic reactions, specifically anaphylaxis or generalized urticaria, rupture of the pseudoaneurysm, and cellulite or abscess [8].

To achieve technical success, the sac of the pseudoaneurysm must be completely thrombosed. Most case series report only a few cases needing a second course of treatment to achieve complete thrombosis. Long-term successful rates are best documented for limb pseudoaneurysms, describing negligible recurrence rates beyond 24 hours after successful initial thrombosis [7,9]. However, any recurrence on follow-up can be treated with further injections [7].

There is no evidence in the literature that anticoagulation affects the efficacy of thrombin embolization [7], so it can be used to successfully treat anticoagulated patients.

## Conclusions

We present a case of a PPAP formed after a transthoracic lung biopsy requiring treatment. After careful research of the literature, we opted for a percutaneous approach given the very peripheric location of the PPAP. Also, since our patient was under anticoagulation, we had to choose a safe embolic agent under this condition. Given the safety profile of thrombin and its efficacy even under anticoagulation, we opted to treat our patient with percutaneous injection of thrombin into the sac of the pseudoaneurysm. Our report of a long-term, successful, minimally invasive treatment of a PPAP with a percutaneous approach using thrombin as an embolic agent reinforces the applicability and efficacy of this kind of treatment approach to PPAP.
